# DNAcycP2: improved estimation of intrinsic DNA cyclizability through data augmentation

**DOI:** 10.1093/nar/gkaf145

**Published:** 2025-03-12

**Authors:** Brody Kendall, Chong Jin, Keren Li, Feng Ruan, Xiaozhong Wang, Ji-Ping Wang

**Affiliations:** Department of Statistics and Data Science, Northwestern University, 633 Clark Street, Evanston, IL 60208, United States; Department of Mathematical Sciences, New Jersey Institute of Technology, 323 Dr Martin Luther King Jr Blvd, Newark, NJ 07102, United States; Department of Mathematics, University of Alabama at Birmingham, 1720 University Blvd, Birmingham, AL 35294, United States; Department of Statistics and Data Science, Northwestern University, 633 Clark Street, Evanston, IL 60208, United States; Department of Molecular Biosciences, Northwestern University, 633 Clark Street, Evanston, IL 60208, United States; Department of Statistics and Data Science, Northwestern University, 633 Clark Street, Evanston, IL 60208, United States

## Abstract

Loop-seq is a pioneering high-throughput assay that enables the simultaneous quantification of intrinsic cyclizability across a large set of DNA fragments. However, the assay’s reliance on biotin-tethered elongated DNA fragments introduces a tethering effect, leading to biased cyclizability measurements. We demonstrate that the current de-biasing technique is inadequate for fully mitigating this bias. To address this, we introduce DNAcycP2, an enhanced software tool that extends the capabilities of our previous platform, DNAcycP. DNAcycP2 incorporates a novel data augmentation approach to more effectively eliminate biotin tether bias, yielding more accurate estimates of intrinsic DNA cyclizability. Additionally, DNAcycP2 offers improved computational efficiency and expands accessibility through a newly developed R package alongside its existing Python package and web server, ensuring broader utility for the research community.

## Graphical Abstract

**Graphical Abstract:**
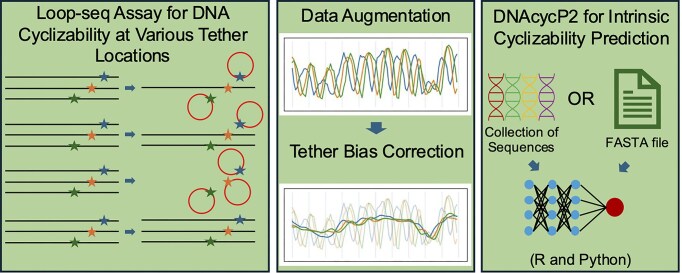

## Introduction

DNA bendability is one of the pivotal aspects of DNA mechanics, playing a critical role in numerous biological processes ranging from DNA packaging and gene expression to DNA replication, repair, and recombination. Across all eukaryotic species, a fundamental structure known as the nucleosome, consisting of 147 bp of DNA wrapped around a histone octamer, is ubiquitous throughout the genome, exerting profound regulatory effects on chromosome functions [1, 2]. These nucleosomes are further organized into higher order structures, forming chromatin fibers within the nucleus [3–5]. Bendability has long been recognized as one of the key factors governing the formation of nucleosomes and chromatin fibers [6–8]. In the process of transcription, cells continuously recruit various transcription factors to bind to DNA, typically in the promoter regions, forming DNA–protein complexes that cooperate with nucleosomes to regulate gene expression [9–11]. In CRISPR–Cas9 genome editing, the Cas9 protein must sharply bend and undertwist DNA on protospacer adjacent motif (PAM) binding, thus, DNA bendability of target site may also correlate with cleavage efficiency [12]. In the field of synthetic biology, by understanding the bending and looping properties of DNA, researchers can design synthetic DNA constructs with desired structural attributes for various biotechnological applications [13, 14]. Thus, quantification of DNA bendability has been highly desirable for us to understand the relationship between DNA mechanics and its various functions.

Decades of effort have led to a comprehensive understanding of DNA bendability through theoretical or experimental approaches [15–22]. But it was not until very recent that massive DNA fragments can be measured for bendability simultaneously through high-throughput sequencing using the loop-seq assay developed by Basu *et al.* [23, 24]. The loop-seq assay quantifies the DNA cyclizability by taking the log ratio of the proportion of a given DNA fragment species in the loop-seq library that formed loops over that in the control library. Although loop-seq measures the propensity to form loops of DNA fragments of the given length, i.e. 50 bp, the resulting intrinsic cyclizability score is mainly determined by the overall intrinsic DNA bendability of the sequence at the given length. Indeed numerous known results that link to DNA bendability have been confirmed using the DNA cyclizability score [23, 25]. Thus, the DNA cyclizability can be regarded as a proxy measure for DNA bendability. However, a loop-seq assay can be costly, and the cyclizability score from different libraries can only be compared using an additive library-specific constant, so a computational method that allows for calculating cyclizability score for any single sequence is highly desirable.

DNAcycP was the first software tool developed for prediction of DNA intrinsic cyclizability based on a deep-learning architecture that achieves high accuracy [25]. DNAcycP has two forms, a free Python package and a web server for real-time online prediction. Several other tools have been published for this purpose and all achieved comparable high accuracy [26–28]. Training of these tools entirely depends on the reported intrinsic cyclizability value from the loop-seq data. The loop-seq assay requires attaching a biotin tether to the inside of the elongated DNA fragment (with adapter and single-strand overhang included in the two ends of the insert DNA). The attaching position of the biotin tether has a dramatic effect on the measured cyclizability. In Basu *et al.*’s work [23], a theoretical model was proposed in which the measured DNA cyclizability is formulated as the convolution of intrinsic cyclizability and a bias term related to biotin position, where the latter follows a sine curve with a periodicity equal to DNA helix repeat length, and the biotin attaching position dictates the relative phase angle. A de-biasing approach was proposed to estimate the intrinsic DNA cyclizability to remove the tether effect. In this paper, we demonstrate that the called intrinsic DNA cyclizability score from the above work still has an undesirable residual biotin effect, and consequently the predicted DNA cyclizability from all existing tools carries the inherent biotin bias. We present the DNAcycP2 software tool with an added R package for broader accessibility (in addition to a Python package and web server) that implements a novel method based on data augmentation for removing biotin bias for the estimation of intrinsic DNA cyclizability. We show the robust and competitive performance of the new method using real and simulated data.

## Materials and methods

### Experimental data

We consider four loop-seq datasets presented in [23]: the Nucleosome, Random, Tiling, and ChrV libraries. Each library contains the measured raw cyclizability values at biotin tether locations $n$ = 26, $n$ = 29, and $n$ = 31 along with the intrinsic cyclizability score ${{C}_0}$ value calculated using Equation (1) (see below) for each 50-bp sequence. The Nucleosome library consists of 19 907 sequences corresponding to fragments immediately to the left or right of the dyads of the unique nucleosomes in the *Saccharomyces cerevisiae* SacCer2 genome from [29]. The Random library consists of 12 472 sequences generated randomly, with each nucleotide equally likely to occur at any position in each sequence. The Tiling library consists of 82 368 sequences selected from 576 genes in the *S. cerevisiae* genome. For each gene, a 1044-bp region was defined surrounding the +1 nucleosome dyad (dyad position ranging from −601 to +442 in the upstream to downstream direction), and 50-bp fragments were selected from this region at 7-bp resolution. The ChrV library consists of 82 404 sequences selected across the entire length of *S. cerevisiae* chromosome V at 7-bp resolution.

### New method to estimate DNA intrinsic cyclizability and DNAcycP2

All existing approaches for prediction of DNA cyclizability assumed the reported intrinsic DNA cyclizability metric, i.e. ${{C}_0}$ from [23] to be the ground truth. By examining the DNA sequence AA/TT/AT/TA motifs in the most and least cyclizable sequences based on ${{C}_0}$ values, and the predicted intrinsic cyclizability scores from existing tools over the nucleosome region, we first show that pronounced biotin bias effect that phases with DNA rotational angle remains in the intrinsic DNA cyclizability score. To dissect the biotin location-specific bias, we trained three independent deep-learning models based on the normalized raw cyclizability scores ${{C}_n}$ for biotin location $n$ = 26, $n$ = 29, and $n$ = 31, respectively, using the identical architecture as DNAcycP [25], except that the model was trained based on the forward strand only (Supplementary Fig. S1A–C). As ${{C}_n}$ carries the inherent biotin bias effect, by applying the trained models to predict ${{C}_n}$ values on the yeast genome, we obtained the base-pair resolution of the augmented ${{C}_n}$ scores that allow us to verify the theoretical model for biotin bias proposed by Basu *et al.* We further propose a local smoothing method with window size 10.4 bp based on the augmented ${{C}_n}$ values to debias the biotin effect. The resulting smoothed ${{C}_n}$ values were further averaged to yield the improved estimate of intrinsic cyclizability on the yeast genome. Lastly using the resulting smoothed intrinsic cyclizability score we re-trained the DNAcycP model and present an updated version named DNAcycP2.

## Results

### Verification of biotin effect model in loop-seq through data augmentation

The biotin location in loop-seq has significant impact on observed cyclizability. It was observed that at biotin position $n$ = 26, the most and least cyclizable DNA sequences both show ∼10 bp periodic, but antiphase A/T content patterns along the sequence [23]. In contrast at $n$ = 31, the most and least cyclizable sequences show the opposite A/T phasing pattern. The contradicting results suggest that the biotin tether location has a major effect on the observed cyclizability value, and this tether effect defined by biotin location $n$ may follow a periodic form with periodicity coinciding with the helical repeat length. Basu *et al.* proposed a theoretic model to account for biotin effect:


(1) \begin{equation*}{{C}_n}\left( i \right) = {{C}_0}\left( i \right)\ + {{{{A}}}_n}\left( i \right)*\sin \left( {\frac{{2\pi n}}{{10.4}} + {{\phi }_0}\left( i \right)} \right),\end{equation*}

where ${{C}_n}( i )$ is the observed cyclizability of the $i$th sequence with tether location $n$; ${{C}_0}$ is the underlying value of intrinsic cyclizability of interest; and ${{{ { A}}}_n}( i )*\sin ( {\frac{{2\pi n}}{{10.4}} + {{\phi }_0}( i )} )$ measures the tether effect where ${{\phi }_0}$ is sequence-specific and ${{{ A}}_n}$ is sequence-/biotin location-specific. Since 10.4 is the helical repeat length, this model implicitly assumes that the biotin effect is determined by the phase angle of biotin location within the helical turn.

We consider extending this model to the sequence in a chromosome scale. If Equation (1) holds, then for the 50-bp sequence centered at position $l$ of a chromosome, denoted ${{\rm S}}{_l} = {{\rm X}}_{l - 24,\, \cdots,} {{\rm X}}_{l + 25}$, we have


(2) \begin{equation*}{{C}_n}\left( l \right) = {{C}_0}\left( l \right)\ + {{{A}}_n}\left( l \right)*\sin \left( {2\pi n/10.4 + {{\phi }_0}\left( l \right)} \right).\end{equation*}

For the 50-bp sequence shifted 1-bp downstream of ${{{\rm S}}_l}$, i.e. ${{\rm S}}_{l + 1} = {{\rm X}}_{l - 23,\, \cdots,} {{\rm X}}_{l + 26}$, we have


\begin{eqnarray*}{{C}_n}(l + 1) &=& {{C}_0}\left( {l + 1} \right) + {{{\rm A}}_n}\left( {l + 1} \right)\nonumber\\ &&*\,\sin \left( {2\pi n/10.4 + {{\phi }_0}\left( {l + 1} \right)} \right).\end{eqnarray*}

**Figure 1.**
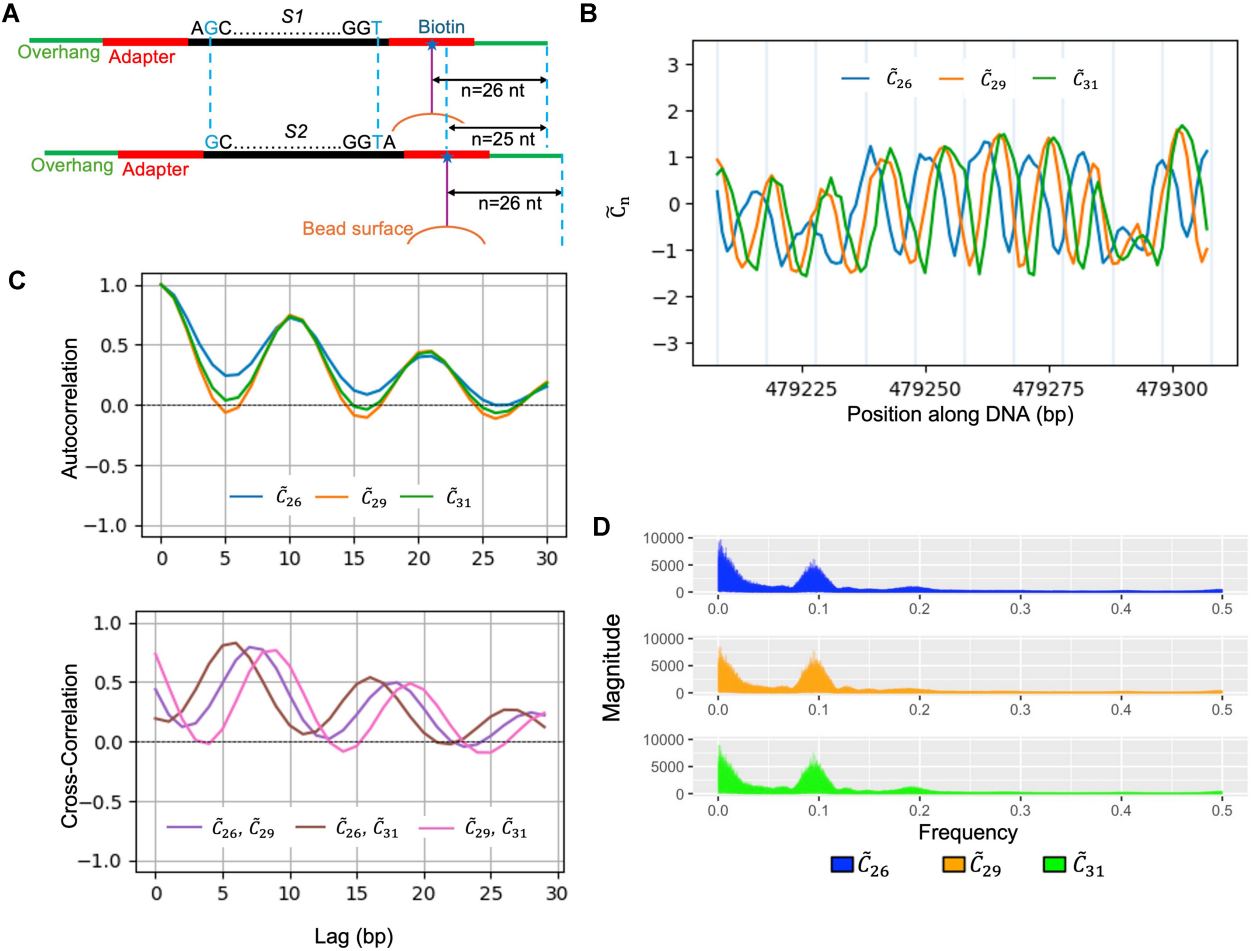
Examination of the underlying assumptions of biotin effect model ${{{\boldsymbol{C}}}_{\boldsymbol{n}}}( {\boldsymbol{i}} ) = {{{\boldsymbol{C}}}_0}( {\boldsymbol{i}} ){\boldsymbol{\ }} + {{{\boldsymbol{{ A}}}}_{\boldsymbol{n}}}( {\boldsymbol{i}} ){\boldsymbol{*}}\sin ( {2{\boldsymbol{\pi n}}/10.4 + {{\phi }_0}( {\boldsymbol{i}} )} )$ (Equation 1). (**A**) Schematic of a 50-bp loop-seq DNA fragment extracted from the genome (with elongated adapter and overhang attached in both ends) with biotin tether location ${\boldsymbol{n}}$ = 26 and the 50-bp loop-seq DNA fragment shifted from the first fragment downstream in the genome by 1 bp with ${\boldsymbol{n}}$ = 26. The DNA rotational angle corresponding to the biotin location ${\boldsymbol{n}}$ = 25 in the first fragment (top panel) will be identical to that corresponding to biotin location ${\boldsymbol{n}}$ = 26 in the second fragment (bottom panel). (**B**) Predicted ${{{\boldsymbol{C}}}_{\boldsymbol{n}}}$ values (${{{\boldsymbol{\tilde{C}}}}_{\boldsymbol{n}}}$) at 1-bp resolution on a given 149-bp region (100 overlapping sequences of length 50) of *S. cerevisiae* chromosome V. (**C**) Auto-correlation of ${{{\boldsymbol{\tilde{C}}}}_{\boldsymbol{n}}}$ (top panel) and cross-correlations of ${{{\boldsymbol{\tilde{C}}}}_{\boldsymbol{n}}}$ between different ${\boldsymbol{n}}$ values (bottom panel) on *S. cerevisiae* chromosome V. The lag ${\boldsymbol{k}}$ of cross-correlation between ${{{\boldsymbol{\tilde{C}}}}_{{\boldsymbol{n}_1}}}$ and ${{{\boldsymbol{\tilde{C}}}}_{{\boldsymbol{n}}_2}}$ is defined as correlation between ${{{\boldsymbol{\tilde{C}}}}_{{\boldsymbol{n}}_1}}( {{\boldsymbol{i}} + {\boldsymbol{k}}} )$ and ${{{\boldsymbol{\tilde{C}}}}_{{\boldsymbol{n}}_2}}( {\boldsymbol{i}} )$, and only ${\boldsymbol{k}}$ = 0, 1, 2, …, is plotted as ${{{\boldsymbol{\tilde{C}}}}_{\boldsymbol{n}}}$ is periodic. (**D**) Fourier power spectrums of ${{{\boldsymbol{\tilde{C}}}}_{\boldsymbol{n}}}$ values on *S. cerevisiae* chromosome V, calculated using the fast discrete Fourier transform method (fft function from R).

From sequence ${{{\rm S}}_{l\ }}$ to ${{{\rm S}}_{l + 1}}$, the DNA helix progresses 1 bp forward, so the biotin location $n$ on sequence ${{{\rm S}}_{l + 1}}$ corresponds to the biotin location $n$−1 on sequence ${{{\rm S}}_{l\ }}$ (Fig. 1A), therefore the corresponding biotin-mediated DNA phase angle inside of the sine function of the two sequences should be the same, i.e. $2\pi n/10.4 + {{\phi }_0}( {l + 1} )=2\pi( {n - 1} )/10.4 + {{\phi }_0}( l )$, or ${{\phi }_0}( {l + 1} )$ = $\ {{\phi }_0}( l )- 2\pi /10.4$. Furthermore since ${{{\rm S}}_{l\ }}$ and ${{{\rm S}}_{l + 1}}$ only differ by 1 bp in sequence composition, we can assume that the true intrinsic ${{C}_0}$ and ${{{A}}_n}$ of ${{{\rm S}}_{l\ }}$ and ${{{\rm S}}_{l + 1}}$ to be very close locally. Thus, the difference of ${{C}_n}$ score for ${{{\rm S}}_l}$ and ${{{\rm S}}_{l + 1}}$ is mainly driven by the sine function in the local scale with a phase difference of −2π/10.4. This result can be further generalized to ${{\phi }_0}( {l + k} )$ = $\ {{\phi }_0}( l )-2k\pi /10.4$ for small $k$, and under which, ${{C}_n}( {l + k} ) \approx {{C}_{n - k}}( l )$ by assuming $\ {{C}_0}$ and ${{{ A}}_n}$ to be close in a small neighborhood of sequence $l$. Plotting of ${{C}_n}$ scores should show a consecutive phase shift for ${{C}_n}$ along the chromosome with step of −2π/10.4 for each base pair moving forward, as well as the fixed phase difference between ${{C}_n}$ and ${{C}_{n - k}}$.

The existing loop-seq data, however, lacks the base-pair resolution that allows to directly investigate this (i.e. only having 7-bp resolution for Tiling/ChrV libraries). To tackle this issue, we trained three deep-learning models (‘‘Materials and methods’’ section), one for each ${{C}_n}$ for $n$ = 26/29/31, respectively, on each of the four libraries: Nucleosome, Random, Tiling, and ChrV. Similar to what was found in other studies, training on the Tiling library yielded the best results (Supplementary Table S1). In the following context, we denote the predicted/augmented ${{C}_n}$ values on the yeast genome based on the Tiling library model as ${{\tilde{C}}_n}$ to distinguish from the observed ${{C}_n}$ values in the raw data. We first note that ${{\tilde{C}}_n}$ is very consistent with the observed ${{C}_n}$ with Pearson correlation ranging from 0.75 to 0.96 (Supplementary Table S1). Secondly for a given $n$, ${{\tilde{C}}_n}( l )$ shows a strong ∼10-bp periodic pattern as a function of chromosome position $l$ along the chromosome in accordance with ${{\phi }_0}( {l + 1} )$ = $\ {{\phi }_0}( l )-2\pi /10.4$ (Fig. 1B). Between different $n$s, a clear progressive phase shift that accords with ${{\tilde{C}}_n}\ ( {l + k} ){\mathrm{\ }} \approx {{\tilde{C}}_{n - k}}\ ( l )$ is shown, e.g. ${{\tilde{C}}_{29}}$ is about 3-bp lagged of ${{\tilde{C}}_{26}}$ (Fig. 1B). These results suggest the DNA rotational angle of the biotin location is the major determinant of the local variation of measured cyclizability as suggested by Equation (2). The auto-correlation plot of ${{\tilde{C}}_n}$ of any given $n$ = 26, 29, and 31 further confirms the ∼10-bp periodicity, whereas the cross-correlation plots of ${{\tilde{C}}_n}$ between different $n$s confirm the observed phase shifts in Fig. 1B, for instance, ${{\tilde{C}}_{26}}$ and ${{\tilde{C}}_{31}}$ are roughly ∼5 bp or a half-period apart, etc (Fig. 1C). Lastly, we performed the Fourier analysis of ${{\tilde{C}}_n}$ on chromosome V of yeast, a clear peak corresponding to periodicity roughly equal to 10.3 bp is consistently observed for all ${{\tilde{C}}_n}$s (Fig. 1D). All these results suggest that the theoretical model by Basu *et al.* is effective to explain the biotin effect in relation to DNA helix phasing.

### Residual biotin tether effect in reported intrinsic cyclizability score

**Figure 2.**
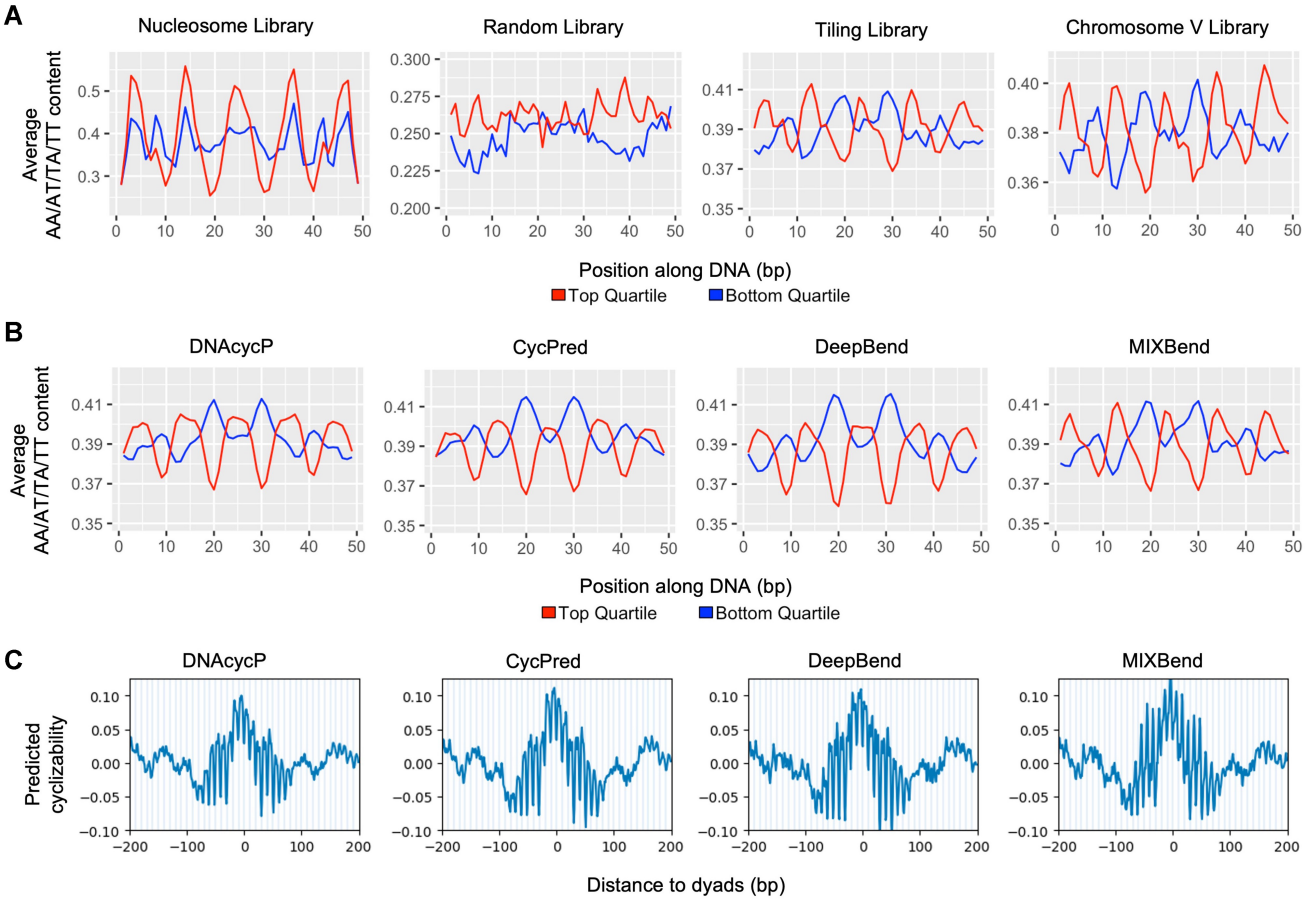
Residual biotin effect in original estimates of intrinsic cyclizability (${{{\boldsymbol{\hat{C}}}}_0}$) reported in [23]. (**A**) Average AA/AT/TA/TT content in sequences corresponding to the top and bottom quartiles of ${{{\boldsymbol{\hat{C}}}}_0}$ values in the four loop-seq libraries: Nucleosome, Random, Tiling, and ChrV (left to right). (**B**) Average AA/AT/TA/TT content in the top and bottom quartiles of predicted intrinsic cyclizability values of every 50-bp sequence on *S. cerevisiae* chromosome V using DNAcycP [25], CycPred [27], DeepBend [26], and MIXBend [28] (left to right). (**C**) Mean predicted intrinsic cyclizability values (normalized) using DNAcycP, CycPred, DeepBend, and MixBend (left to right) aligned at all unique nucleosome dyads in *S. cerevisiae* chromosome V from [29].

Despite the evidence supporting the basic assumptions presented in Equation (1), we noticed significant residual biotin effect remaining in estimated ${{C}_0}$ values from [23], which for convenience, is to be denoted as ${{\hat{C}}_0}$ in the following context (we reserve ${{C}_0}$ for the ground truth of the intrinsic cyclizability). For each of the four libraries, we first selected the sequences corresponding to the top and bottom quartiles of ${{\hat{C}}_0},$ and plotted the average AA/AT/TA/TT dinucleotide frequency along the sequences (Fig. 2A, the pattern of A/T mononucleotide frequency is similar, results not shown). In both Tiling and ChrV libraries the top and bottom quartiles both show strong periodic but antiphase oscillations of AA/TT/TA/TT motifs at ∼10-bp periodicity (Fig. 2A). For the Nucleosome library, as sequences were extracted at fixed locations relative to the dyad of nucleosomes from [29], sequences from both quartiles are expected to have the same or similar rotational angle in the helix turn. Consequently, we observe the well-known periodic AA/TT/TA/AT motifs with synchronized phasing pattern [29, 30]. The higher AA/TT/TA/AT amplitude corresponds to higher cyclizability, confirming the well-known fact that periodic AA/TT/TA/AT motif facilitates bending of DNAs [31–34]. For the Random library, the AA/AT/TA/TT frequency in the top/bottom quartiles is very noisy, lacking a pattern. This is not surprising as the ratios between ${{{A}}_n}$s in Equation (1) were tuned to minimize the resulting periodic pattern of A/T content in the top 1000 most cyclizable sequences when solving the linear equation for ${{C}_0}$ in [23]. Apparently, the choice of ${{{A}}_n}$ ratios that minimized the A/T periodicity in the Random library failed to adequately remove the biotin tether bias in other libraries.

Next, we examined the predicted intrinsic cyclizability from four existing deep-learning models, including DNAcycP [25], CycPred [27], DeepBend [26], and MIXBend [28], all trained on the reported ${{\hat{C}}_0}$ values. (Note: as [28] does not provide a final model or software tool, we used the provided code to train a model on the Tiling library, from here on referred to as MIXBend). The AA/TT/TA/AT content for the 50 bp sequences extracted from yeast ChrV library corresponding to the top and bottom quartiles of predicted intrinsic cyclizability show similar antiphase oscillation patterns as observed in Tiling and ChrV libraries (Fig. 2B). Lastly, we plotted the average of normalized predicted intrinsic cyclizability score from these tools over all unique nucleosomes on yeast chromosome V from [29], and observe a strong 10 bp periodic pattern that clearly phases with DNA rotational angle (Fig. 2C). These results all suggest that a significant portion of tether bias maintains in the ${{\hat{C}}_0}$ values, which is also carried over to the predicted intrinsic cyclizability score from all the tools trained from it. A new method to debias biotin tether effect is desired to provide more accurate estimation and prediction of DNA intrinsic cyclizability.

### New method to debias biotin tether effect using augmented data

Equation 1 is elegant and effective to describe the dependence of biotin tether effect on its location relative to DNA rotational angle, while posing some uncertainty in modeling the amplitude parameter ${{{A}}_n}$. For a given sequence and a biotin location $n$, as there is only one measurement of ${{C}_n}$ in the loop-seq data, ${{{A}}_n}$ cannot be estimated directly without making further assumptions. Basu *et al.* noticed that accounting for some dependence between ${{{A}}_n}$s allowed to improve the estimates of ${{C}_0}$. Namely, they defined ${\rm Q}( f ) = {{C}_{26}} + f*{{C}_{31}}$ and found that a value of $f$ = 0.7 minimized the Fourier power corresponding to period ∼10 bp of the A/T signal for the sequences with the top 1000 $Q( f )$ values in the Random library. To incorporate this into their model, they set the ratio ${{{A}}_{26}}$/${{{A}}_{31}}$ to be 0.7 for all sequences (and by linear interpolation, ${{{A}}_{26}}$/${{{A}}_{29}}$ to be equal to 0.82). Under this, for each sequence, solutions to ${{C}_0}$, ${{{A}}_n},$ and ${{\phi }_0}$ can be solved perfectly through linear equations, roughly ${{\hat{C}}_0} \approx \frac{{\frac{{{{C}_{26}}}}{{0.7}} + \ {{C}_{31}}}}{{1 + \frac{1}{{0.7}}}}$ (due to nearly perfect antiphase of biotin effect of ${{C}_{26}},\ {{C}_{31}}$). Clearly ${{C}_{26}}$ has larger weight than ${{C}_{31}}$ in the estimated ${{C}_0}$ value. Comparing the AA/TT/TA/AT plots from the top and bottom quartiles based on ${{\hat{C}}_0}$, ${{C}_{26,}}\ {{C}_{29,\ }}\ {{C}_{31}}$ values, we found the phasing pattern of AA/TT/TA/AT motif in the most/least cyclizable sequences based on ${{\hat{C}}_0}$ most resembles that from ${{C}_{26}}$ (Supplementary Fig. S2). Further, we plotted the average DNAcycP predicted intrinsic cyclizability scores over all unique nucleosomes on the entire yeast genome and observed that its phasing pattern matches that of ${{\tilde{C}}_{26}}$ (Supplementary Fig. S3). These results suggest that how to model ${{{A}}_{\mathrm{n}}}$ for different $n$s may be a crucial step in debiasing biotin effect.

**Figure 3.**
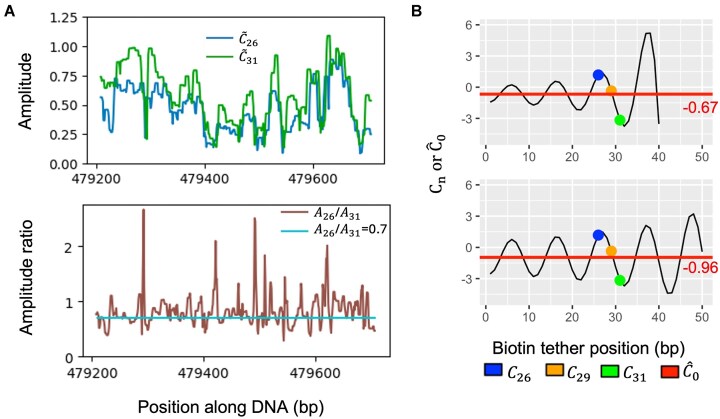
Examination of the relationship between amplitude (${{{\boldsymbol{{A}}}}_{\boldsymbol{n}}}$) and tether location (${\boldsymbol{n}}$) in the biotin effect model in Equation (1) and its impact on estimation of ${{{\boldsymbol{C}}}_0}$. (**A**) Estimated amplitude (top panel) and estimated amplitude ratios (bottom panel) over a 549-bp region of *S. cerevisiae* chromosome V, in the elongated region of Fig. 1B. The constant ratio ${{{\boldsymbol{{A}}}}_{26}}$/${{{\boldsymbol{{A}}}}_{31}}$ = 0.7 represents the assumption made in [23]. The amplitude for a given ${\boldsymbol{n}}$ at location ${\boldsymbol{l}}$ is approximated by half range of ${{{\boldsymbol{\tilde{C}}}}_{\boldsymbol{n}}}$ values (unnormalized) in the region [${\boldsymbol{l}}$−5, ${\boldsymbol{l}}$+5]. (**B**) An example showing different assumptions of the ratio between ${{{\boldsymbol{{A}}}}_{\boldsymbol{n}}}$s may give dramatically different estimates of ${{{\boldsymbol{C}}}_0}.$ The ${{{\boldsymbol{C}}}_{26,}}{\boldsymbol{\ }}{{{\boldsymbol{C}}}_{29,{\boldsymbol{\ }}}}$ and ${\boldsymbol{\ }}{{{\boldsymbol{C}}}_{31}}$ values of a randomly selected sequence from Tiling library are 1.18, −0.36, and −3.17, respectively. The plotted are the ${\boldsymbol{\ }}{{{\boldsymbol{C}}}_{\boldsymbol{n}}}$ values as a function of biotin location ${\boldsymbol{n}}$ under two different assumptions: ${{{\boldsymbol{{A}}}}_{26}}$/${{{\boldsymbol{{A}}}}_{31}}$ = 0.7 (top panel) and ${{{\boldsymbol{{A}}}}_{26}}$/${{{\boldsymbol{{A}}}}_{31}}$ = 0.9 (bottom panel). In both cases ${{{\boldsymbol{{A}}}}_{26}}$/${{{\boldsymbol{{A}}}}_{\boldsymbol{n}}}$ was calculated using linear interpolation/extrapolation as in [23]: ${{{\boldsymbol{A}}}_{\boldsymbol{26}}}{\boldsymbol{\ }}/{{{\boldsymbol{A}}}_{\boldsymbol{n}}}{\boldsymbol{\ }} = 1 - {\boldsymbol{\ }}\frac{{( {{\boldsymbol{n}} - 26} ){\boldsymbol{*}}( {1 - \frac{{{{{\boldsymbol{A}}}_{26}}}}{{{{{\boldsymbol{A}}}_{31}}}}} )}}{5}{\boldsymbol{\ }}$ for other ${\boldsymbol{n}}$s. Both curves fit the observed data perfectly while the resulting estimates of ${{{\boldsymbol{C}}}_0}{\boldsymbol{\ }}$ by solving Equation (1) are −0.67 and −0.94, respectively.

The base pair resolution of the ${{\tilde{C}}_n}$ values revealed that the major variability of observed ${{C}_n}$ is attributed to biotin effect or to the relatively large value of ${{{A}}_{{n}}}$ in the model (Fig. 1B). Locally, as ${{\tilde{C}}_n}$ roughly evolves according to ${{C}_n}( l ) = {{C}_0}( l )\ + {{{A}}_n}( l )*\sin ( {2\pi n/10.4 + {\phi }_0}( l ) )$ along the chromosome sequence, and ${{\phi }_0}( {l + 1} )= {{\phi }_0}( l )-2\pi /10.4$ we can approximate ${{{A}}_{{n}}}$ at position $l$ by half range of ${{\tilde{C}}_n}$ values in the region of $l$ ± 5 bp, assuming ${{C}_0}$ and ${{{A}}_{{n}}}$ remain relatively constant in this local region. We expanded the example region presented in Fig. 1B and calculated the approximate ${{{A}}_{26}}$ and ${{{A}}_{31}}$ at each location based on ${{\tilde{C}}_{26}}$ and ${{\tilde{C}}_{31}}$, and then the approximate ratio of ${{{A}}_{26}}$ over ${{{A}}_{31}}$ (Fig. 3A). Both ${{{A}}_{26}}$, ${{{A}}_{31}}$ and their approximate ratio vary substantially in the long range. To illustrate how the specified ratios may impact the estimates of ${{C}_0}$, $\phi$, and ${{{A}}_n}$, we select one arbitrary sequence from loop-seq Tiling library, of which the observed ${{C}_{26,}}\ {{C}_{29,\ }}$ and $\ {{C}_{31}}$ values are 1.18, −0.36, and −3.17, respectively (Fig. 3B). If, we assume ${{{A}}_{26}}$/${{{A}}_{31}}$ = 0.7, and ${{{A}}_{26}} / {{{A}}_{\mathrm{n}}} = 1 - \ \frac{{( {n - 26} )*0.3}}{5}$ (linear interpolation for $n$ = 31), then ${{\hat{C}}_0}$ is −0.67 based on Equation (1). However, if we change our assumption to ${{{A}}_{26}}$/${{{A}}_{31}}$ = 0.9, then ${{\hat{C}}_0}$ becomes −0.96. Since we only have three ${{C}_n}$ values per sequence, it is infeasible to determine an estimate for ${{C}_0}$, ${{\phi }_0}$, and ${{{A}}_n}$ for each $n$ without some assumptions on ${{{A}}_n}$.

**Figure 4.**
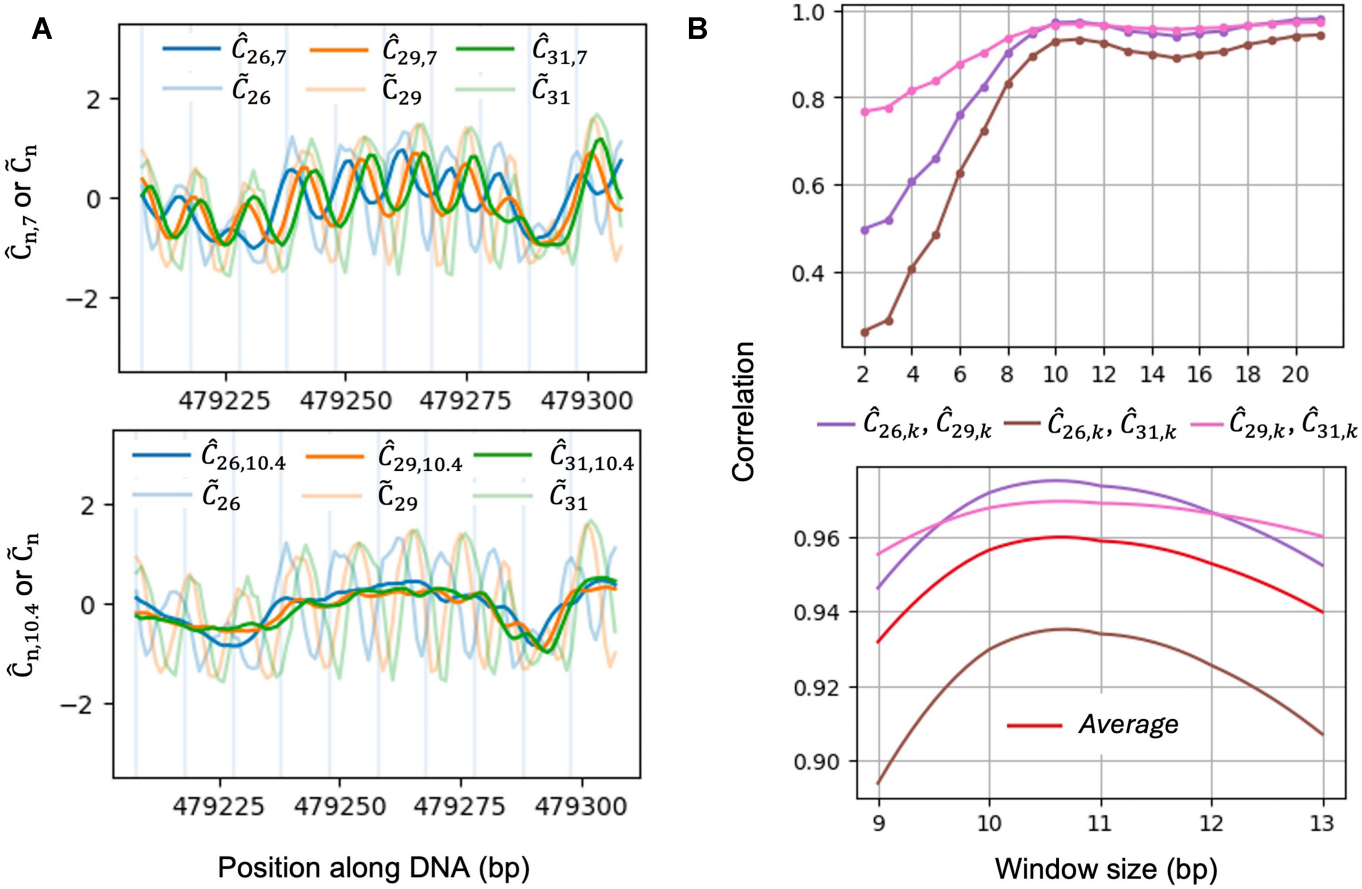
The moving average of the predicted/augmented ${{{\boldsymbol{C}}}_{\boldsymbol{n}}}$ values (i.e. ${{{\boldsymbol{\tilde{C}}}}_{\boldsymbol{n}}}$) at different window size ${\boldsymbol{k}}$. (**A**) Smoothed ${{{\boldsymbol{\tilde{C}}}}_{\boldsymbol{n}}}$ over window size ${\boldsymbol{k}}{\mathrm{\ }}({{{\boldsymbol{\hat{C}}}}_{{\boldsymbol{n}},{\boldsymbol{k}}}}$) using Equation (3) for ${\boldsymbol{k}}$ = 7 bp (top panel), and *k* = 10.4 bp (bottom panel) in the example region from *S. cerevisiae* chromosome V in Fig. 1B. (**B**) Correlation between ${{{\boldsymbol{\hat{C}}}}_{{\boldsymbol{n}},{\boldsymbol{k}}}}$ values at different ${\boldsymbol{n}}$s as a function of window size k. The top panel has a wide range of integer values of ${\boldsymbol{k}}$ from 2 to 21 bp, while the bottom panel has a zoomed in, more refined set of ${\boldsymbol{k}}$ values in the range of 9–13 bp.

To tackle this issue without making strong assumptions of ${{A}_n}$, we propose a novel approach based on the moving average method of the augmented ${{C}_n}$ values on the yeast genome. First, we expect the true intrinsic cyclizability based on the 50-bp sequence to evolve smoothly along the chromosome as for each base-pair move, the majority of the sequence is preserved. Secondly, with the convincing evidence shown above that the biotin effect follows a sine curve with ∼10-bp periodicity, the average ${{\tilde{C}}_n}$ value across a ∼10-bp region tends to cancel off the biotin effect, assuming ${{A}_n}$ to remain relatively constant in the local region. At the $l$th position of the chromosome, we define ${{\hat{C}}_{n,k}}( l )$ as the smoothed ${{\tilde{C}}_n}$ value over a $k$-size window as follows:


(3) \begin{eqnarray*}{{\hat{C}}_{n,k}} (l) = \left\{\begin{array}{l{@}{\quad}l}\frac{1}{k}\sum \limits_{j = l - \frac{{k - 1}}{2}}^{l + \frac{{k - 1}}{2}} {\tilde{C}}_{n}(j),\!\!\!\!& \ k\ {\rm is\ an\ odd\ integer},\\\frac{1}{{2k}}\left( {{{{\tilde{C}}}_n}\left( {l - \frac{k}{2}} \right) + {{{\tilde{C}}}_n}\left( {l + \frac{k}{2}} \right)} \right) & \\+ \frac{1}{k}\sum \limits_{j = l - \frac{k}{2} + 1}^{l + \frac{k}{2} - 1} {\tilde{C}}_{n} (j),\!\!\!\!& k\ {\rm is\ an\ even\ integer},\\\frac{{w}_{1}*{\hat{C}}_{n,k1} (l) + {w}_{2}*{\hat{C}}_{n,k2} (l)}{{w}_{1} + {w}_{2}},& {\rm otherwise},\end{array}\right. \end{eqnarray*}}

where ${{k}_1}$ and ${{k}_2}$ are the two closest integers to $k{\mathrm{\ }}({{k}_1} < \ {{k}_2}$), ${{w}_1} = \frac{1}{{k - {{k}_1}}}$, and ${{w}_2} = \frac{1}{{{{k}_2} - k}}$. We choose a $k$ that can remove the local cyclic pattern of ${{\tilde{C}}_n}$ due to biotin bias, and henceforth yield consistent estimate of ${{C}_0}$ across different $n$s, given which, we prefer a relatively small $k$ to minimize the loss of resolution of the signal. We tried various values for the smoothing parameter $k$ and plotted $\ {{\hat{C}}_{n,k}}$ for $k$ = 7 vs $k$ = 10.4 in Fig. 4A as an illustration. For the same region presented in Fig. 1B, $k$ = 7 did not completely remove the phasing biotin bias; in contrast $k$ = 10.4 not only removed most of the local phasing pattern from each $\ {{\tilde{C}}_n}$, but also generated highly consistent pattern of $\ {{\hat{C}}_{n,k}}$ for different $n$s. To further quantify the consistency, we plotted the correlation of $\ {{\hat{C}}_{n,k}}$between $n$ = 26/29/31 at different smoothing window size $k$ (Fig. 4B), and further zoomed in from $k$ = 9 to 13. The average pairwise correlation reached the highest when $k$ is between 10.4 and 11, so we choose $k$ = 10.4 through the following context.

### Simulation study

To further investigate whether the smoothed ${{C}_n}$ values can be used to estimate the true underlying $\ {{C}_0}$, we conducted three simulation studies by generating the ${{C}_n}$ values for $n$ = 26, 29, and 31 according to the theoretical model in Equation (1) as follows:

-
${{C}_{n}}( l ) = {{C}_0}( l )\ + \ {{A}_n}( l )*\sin ( {\frac{{2\pi n}}{{10.4}} + \phi ( l )} ) + \ \varepsilon ( l )$ for ${{A}_{26}}$/${{A}_{31}} = 0.7$ and ${{A}_{26}}$/${{A}_{29}} = 0.82$,
-
${{C}_n}( l ) = {{C}_0}( l )\ + \ {{A}_n}( l )*\sin ( {\frac{{2\pi n}}{{10.4}} + \phi ( l )} ) + \ \varepsilon ( l )$ for ${{A}_{26}}$/${{A}_{31}} = \frac{1}{{0.7}}$ and ${{A}_{26}}$/${{A}_{29}} = \frac{1}{{0.82}}$,
-
${{C}_n}( l ) = {{C}_0}( l )\ + \ {{A}_n}( l )*\sin ( {\frac{{2\pi n}}{{10.4}} + \phi ( l )} ) + \ \varepsilon ( l )$ for ${{A}_{31}} = a*{{A}_{26}}$ and ${{A}_{29}} = b*{{A}_{26}}$.

We generated one set of 100 000 ground-truth ${{C}_0}$ values using a random-walk approach where the pace of each step follows a normal distribution: ${{C}_0} ( {l + 1} )\ = {{C}_0}( l ) + N( {0,\ 1/144} )$. We maintain the ${{C}_0}$ value in the range of [−2, 2] to prevent over-drift of the value. If ${{C}_0}\ ( {l + 1} )$ gets outside of the range, we re-generate ${{C}_0}( {l + 1} )$by moving from ${{C}_0}( l )$ to the opposite direction with the same random pace generated (same approach applied to other parameters below). The baseline ${{A}_{26}}$ values for each setting were generated separately using a similar random-walk approach, namely, ${{A}_{26}}( {l + 1} ) = {{A}_{26}}( l ) + N( {0,\ 1/25} )$ such that ${{A}_{26}}( l ) \in$ [0.1, 3]. For simulation (iii), we allowed the ratios of ${{A}_n}$s to stochastically evolve: ${{A}_{31}} = a*{{A}_{26}}$ and ${{A}_{29}} = b*{{A}_{26}}$, where $\log ( a )$ and $\log ( b )$ were simulated independently by a random walk with step function following $\ N( {0,\ 1/100} )$ such that a, b $ \in$ [0.2, 1.5]. For each setting, we imposed a noise term $\varepsilon \ ( l )\ \sim\ N( {0,\ 1/16} )$ on top of true intrinsic cyclizability and the biotin effect. Lastly, the parameter $\phi (l)$ was initialized randomly and the rest calculated to mimic the phase angle progression in DNA sequence, i.e. $\phi ( {l + 1} )\ = \phi ( l ) - 2\pi /10.4$.

**Figure 5.**
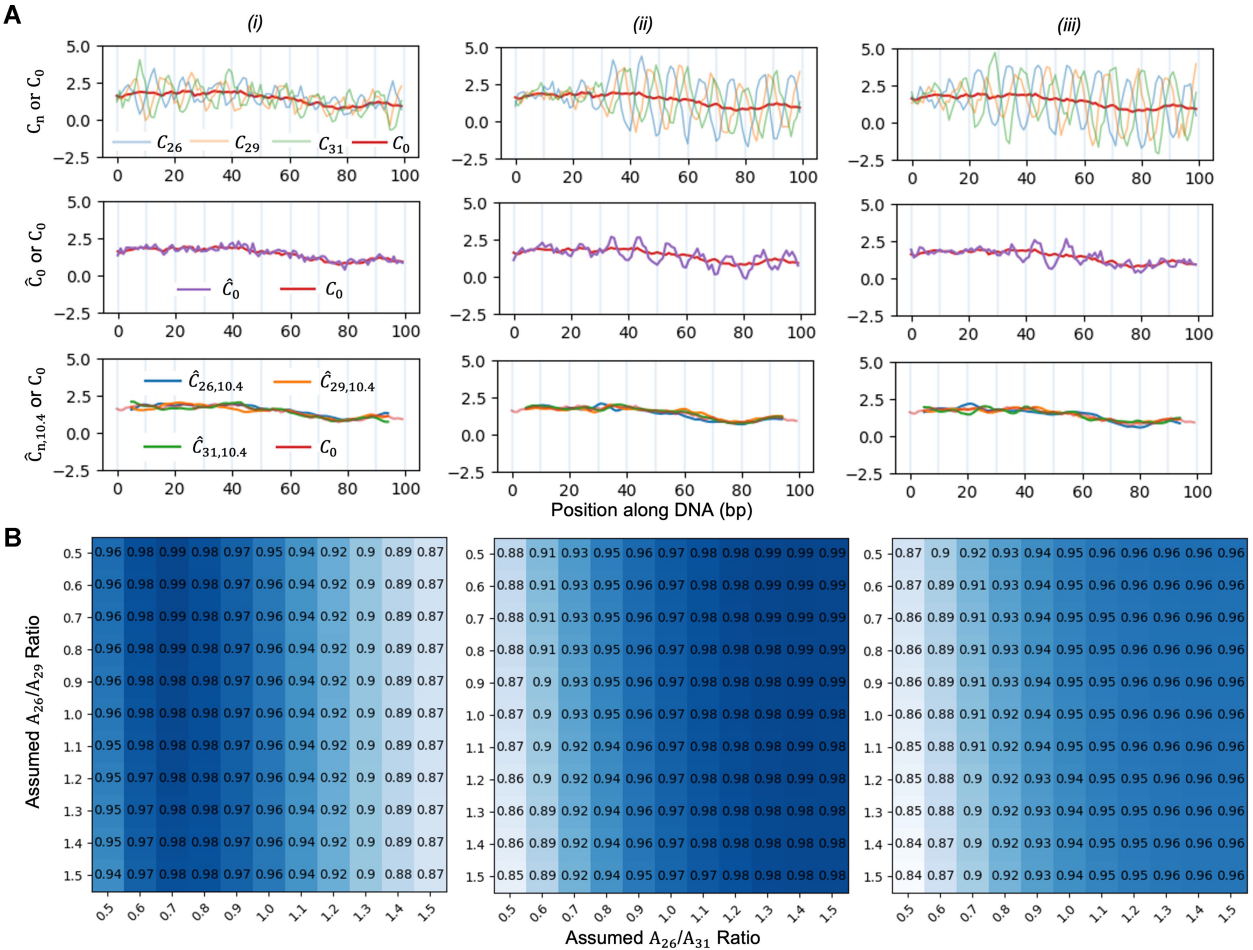
Simulation study. (**A**) Simulated ${\boldsymbol{\ }}{{{\boldsymbol{C}}}_{\boldsymbol{n}}}$ values and the intrinsic cyclizability (${{{\boldsymbol{C}}}_0}$) (top row), compared with the ${{{\boldsymbol{\hat{C}}}}_0}$ calculated using Equation (1) assuming ${{{\boldsymbol{A}}}_{26}}$/${{{\boldsymbol{A}}}_{31}}$ = 0.7 and ${{{\boldsymbol{A}}}_{26}}$/${{{\boldsymbol{A}}}_{29}}$ = 0.82 (middle row), and ${{{\boldsymbol{\hat{C}}}}_{{\boldsymbol{n}},10.4}}$ values (the smoothed ${\boldsymbol{\ }}{{{\boldsymbol{C}}}_{\boldsymbol{n}}}$ values at ${\boldsymbol{k}}$=10.4) using Equation (3) (bottom row). Plotted are only for values from position 1 to 100 (out of 100 000) for illustration purpose. (**B**) Heatmap plot of correlations between the simulated ground truth ${{{\boldsymbol{C}}}_0}$ values and ${{{\boldsymbol{\hat{C}}}}_0}$ values calculated using Equation (1) with various pairs of fixed values of ${{{\boldsymbol{A}}}_{26}}$/${{{\boldsymbol{A}}}_{31}}$ and ${{{\boldsymbol{A}}}_{26}}$/${{{\boldsymbol{A}}}_{29}}$.

Although we cannot simulate the DNA helix structure, the imposed auto-correlation structure by random walk and the phase angle $\phi ( {l + 1} )\ = \phi ( l ) - 2\pi /10.4$ setting effectively simulated the smooth transition of ${{C}_0}$ and ${{A}_n}$ values and the oscillating pattern of ${{C}_n}$ values dictated by the sine function with the periodicity equal to helix repeat length 10.4 bp (Fig. 5A). We calculated the smoothed ${{C}_n}$ values at $k$ = 10.4, i.e. ${{\hat{C}}_{n,10.4}}$ for each $n$, and then their average, ${{\bar{C}}_{.,10.4}} = \ ( {{{{\hat{C}}}_{26,10.4}} + {{{\hat{C}}}_{29,10.4}} + {{{\hat{C}}}_{31,10.4}}} )/3$. For comparison, we also calculated ${{\hat{C}}_0}$ based on Equation 1 for each setting assuming the$\ {{A}_{26}}$/${{A}_{31}} = 0.7$ and ${{A}_{26}}$/${{A}_{29}} = 0.82$ (Fig. 5A). The correlation between the raw ${{C}_n}$ values, i.e. ${{C}_{26}},\ \ {{C}_{29}},\ {{C}_{31}},$ and ${{C}_0}$ ranges from 0.53 to 0.78 in the three settings, suggesting ${{C}_n}$ carries the intrinsic cyclizability information while the correlation is dampened by the biotin bias and random error (Table 1). For ${{\hat{C}}_0}$ in setting (i) where the ratios were set as the ground truth, i.e. $\ {{A}_{26}}$/${{A}_{31}} = 0.7$ and ${{A}_{26}}$/${{A}_{29}} = 0.82$, the correlation between ${{\hat{C}}_0}$ and ${{C}_0}$ is 0.985, where the nonperfect correlation is due to the random noise. In contrast the correlations between $\ {{\hat{C}}_{n,10.4}}$ and ${{C}_0}$ for $n$ = 26, 29, and 31 in setting (i) are 0.991, 0.989, and 0.987, respectively, suggesting the smoothed cyclizability score at different $n$ values can slightly outperform ${{\hat{C}}_0}$, even when ${{\hat{C}}_0}$ is calculated when the ratios between ${{A}_n}$s are correctly specified. Unsurprisingly, an incorrect specification of ${{A}_n}$ ratios or variable ratios between ${{A}_n}$s as in setting (ii) and (iii) may result in noticeably worse performance of ${{\hat{C}}_0}$, with correlation decayed to 0.93 and 0.91, respectively. In particular, the resulting ${{\hat{C}}_0}$ values may still carry the residual biotin bias, as evidenced by the oscillating pattern along the sequence (Fig. 5A, middle panel). In contrast, $\ {{\hat{C}}_{n,10.4}}$ exhibits robustness to such variations, achieving a correlation of 0.991–0.993 for setting (ii) and 0.991–0.992 for setting (iii) across $n$s. Lastly, ${{\bar{C}}_{.,10.4}}$, the average of $\ {{\hat{C}}_{n,10.4}}$ over $n$s, performs even better than individual $\ {{\hat{C}}_{n,10.4}}$ in every setting, achieving correlation of 0.996 in all three settings (Table 1).

**Table 1.** Simulation study results

	Simulation Setting		
	(i)	(ii)	(iii)
${{{\boldsymbol{C}}}_{26}}$	0.660	0.659	0.661
${{{\boldsymbol{C}}}_{29}}$	0.588	0.727	0.776
${{{\boldsymbol{C}}}_{31}}$	0.528	0.777	0.772
${{{\boldsymbol{\hat{C}}}}_{26,10.4}}$	0.991	0.991	0.991
${{{\boldsymbol{\hat{C}}}}_{29,10.4}}$	0.989	0.993	0.992
${{{\boldsymbol{\hat{C}}}}_{31,10.4}}$	0.987	0.993	0.992
${{{\boldsymbol{\bar{C}}}}_{.,10.4}}$	**0.996**	**0.996**	**0.996**
${{{\boldsymbol{\hat{C}}}}_0}$	0.985	0.929	0.910

Correlations between the simulated ground truth ${{C}_0}$ values and the simulated ${{C}_n}$ values, smoothed ${{C}_n}$ values (${{\hat{C}}_{n,10.4}}$), the average of smoothed ${{C}_n}$ values (${{\bar{C}}_{.,10.4}}$), and ${{\hat{C}}_0}$, the estimated ${{C}_0}$values using Equation 1, respectively for each study (i)–(iii).

To further investigate how incorrect specification of the amplitude ratios affects the performance of ${{\hat{C}}_0}$, we constructed a grid of possible values for ${{A}_{26}}$/${{A}_{31}}$ and ${{A}_{26}}$/${{A}_{29}}$ between 0.5 and 1.5. At each setting of ${{A}_{26}}$/${{A}_{31}}$ and ${{A}_{26}}$/${{A}_{29}}$ values, we calculated the correlation between ${{\hat{C}}_0}$ and ${{C}_0}$ and presented the results in a heatmap (Fig. 5B). In all settings, ${{\hat{C}}_0}$ is insensitive to ${{A}_{26}}$/${{A}_{29}}$ as expected, as roughly ${{\hat{C}}_0}$ is determined by ${{C}_{26}}$ and ${{C}_{31}}$ since the biotin effect is antiphase according to Equation (1). For settings (i) and (ii), the performance ${{\hat{C}}_0}$ decays as the specified ratio ${{A}_{26}}$/${{A}_{31}}$ further deviates from the true value. Since the true amplitude ratios are not constant in setting (iii), defining any fixed set of amplitude ratios is incorrect, resulting in consistently lower correlation values in the corresponding heatmap. In summary, we conclude that the residual biotin effect in ${{\hat{C}}_0}$ in the loop-seq data may be due to inaccurate specification of the ratios of ${{A}_n}$s, and the smoothed ${{C}_n}$ values, ${{\hat{C}}_{n,10.4}}$, and their average, ${{\bar{C}}_{.,10.4}}$, can not only robustly remove the biotin bias but also improve the precision of the estimate of ${{C}_0}$ due to averaging.

To investigate the sensitivity of our local smoothing method to different levels of noise and smoothing parameters, we conducted an additional sensitivity analysis. We varied the random walk variances for ${{C}_0}$, ${{A}_n}$, $\log ( a )$, and $\log ( b )$, as well as the error variance, while keeping the smoothing window size $k$ fixed at 10.4 bp. The results presented in Supplementary Table S2 demonstrate the robustness of our method across a range of parameter values. Most notably, the correlation between the estimated intrinsic cyclizability (${{\bar{C}}_{.,10.4}}$) and the true intrinsic cyclizability (${{C}_0}$) remained consistently high (above 0.99) even when the noise levels or the random walk variance parameters were significantly raised. This finding provides further support for the effectiveness of our local smoothing method in accurately estimating intrinsic cyclizability, even in the presence of substantial noise.

### New method to estimate intrinsic DNA cyclizability on the yeast genome

**Figure 6.**
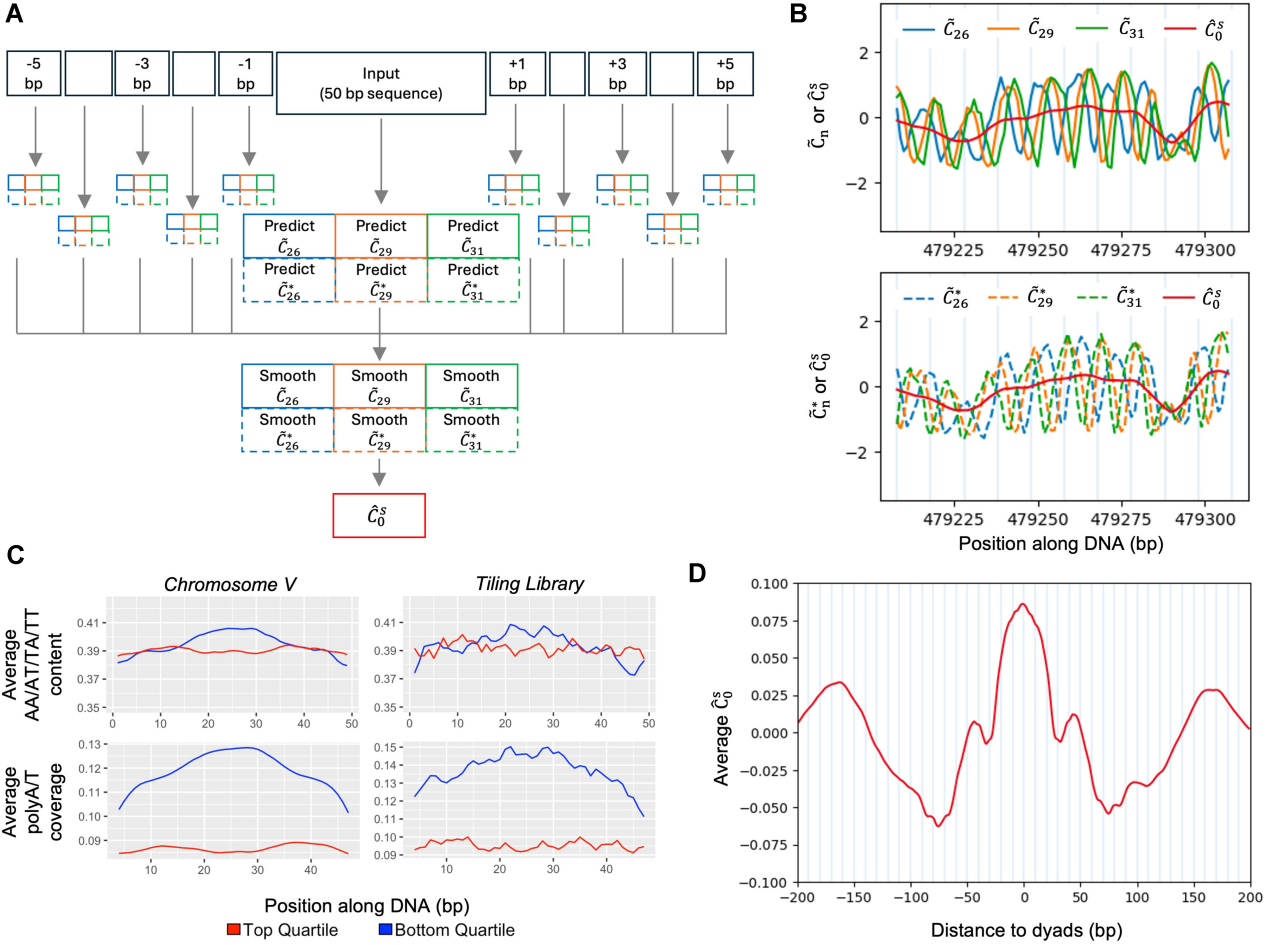
Proposed new estimate of intrinsic cyclizability (${\boldsymbol{\hat{C}}}_0^{\boldsymbol{s}}$). (**A**) Computation workflow. We predict the ${{{\boldsymbol{C}}}_{\boldsymbol{n}}}$ values for ${\boldsymbol{n}}$ = 26, 29, and 31 on both the forward (${{{\boldsymbol{\tilde{C}}}}_{\boldsymbol{n}}}$) and reverse complement (${\boldsymbol{\tilde{C}}}_{\boldsymbol{n}}^{\boldsymbol{*}}$) strands for every 50-bp sequence on the yeast genome. Then, we independently smooth each of these six sets of values using Equation (3) with window size ${\boldsymbol{k}}$= 10.4 (${{{\boldsymbol{\hat{C}}}}_{{\boldsymbol{n}},10.4}}$ and ${\boldsymbol{\hat{C}}}_{{\boldsymbol{n}},10.4}^{\boldsymbol{*}}$). ${\boldsymbol{\hat{C}}}_0^{\boldsymbol{s}}$ is defined as the mean of the six smoothed values. (**B**) ${\boldsymbol{\hat{C}}}_0^{\boldsymbol{s}}$ compared with ${{{\boldsymbol{\tilde{C}}}}_{\boldsymbol{n}}}$ (top panel) and ${\boldsymbol{\tilde{C}}}_{\boldsymbol{n}}^{\boldsymbol{*}}$ (bottom panel) on the example region of yeast chromosome V in Fig. 1B. (**C**) Average AA/AT/TA/TT content (top row) and average poly (dA:dT) coverage (the proportion of sequences which have a stretch of A or T of length 4 or above at a given position) (bottom row) in all 50-bp sequences corresponding to top and bottom quartiles of ${\boldsymbol{\hat{C}}}_0^{\boldsymbol{s}}$ values on yeast chromosome V (left column) and Tiling library (right column). (**D**) Mean ${\boldsymbol{\hat{C}}}_0^{\boldsymbol{s}}$ values aligned at all nucleosome dyads in the full genome of *S. cerevisiae*.

We propose a new estimator of intrinsic cyclizability score, to be denoted $\hat{C}_0^s$ to distinguish from the originally reported intrinsic cyclizability score ${{\hat{C}}_0}$ from [23], as follows:


(4) \begin{eqnarray*} &&\hat{C}_0^s\left( l \right)\nonumber\\ &&= \frac{{{{{\hat{C}}}_{26,10.4}}\left( l \right) + \ {{{\hat{C}}}_{29,10.4}}\left( l \right) + {{{\hat{C}}}_{31,10.4}}\left( l \right) + \hat{C}_{26,10.4}^*\left( l \right) + \hat{C}_{29,10.4}^*\left( l \right) + \hat{C}_{31,10.4}^*\left( l \right)}}{6},\quad \nonumber\\ \end{eqnarray*}}

where the ${{\hat{C}}_{n,10.4}}\ ( l )$ and $\hat{C}_{n,10.4}^*\ ( l )$ are the smoothed augmented ${{C}_n}$ values at location $l$ with smoothing parameter $k$ = 10.4 for the forward and reverse complement strands, respectively, on the yeast genome (Fig. 6A). As an illustration, we plotted $\hat{C}_0^s$ in the example region presented in Fig. 1B, along with the augmented ${{\tilde{C}}_n}$ values from the forward and reverse strands (Fig. 6B). Regardless that the ${{C}_n}$ curves from the two strands may have a phase difference, the resulting $\hat{C}_0^s$ values were able to consistently capture the main trend of both strands, while successfully removing the phasing pattern of individual ${{C}_n}$ curves.

To investigate whether the new estimate of intrinsic cyclizability score $\hat{C}_0^s$ has successfully removed the biotin effect, we extracted the 50-bp sequences from yeast chromosome V that represent the top and bottom quartiles of $\hat{C}_0^s$ score and plotted the AA/TT/AT/TA frequency along the sequence. Indeed, the periodic pattern of AA/AT/TA/TT disappeared for both quartiles (Fig. 6C, top-left panel) (in contrast to the antiphasing periodic pattern observed in the top/bottom quartiles based on the original ${{\hat{C}}_0}$ values in Fig. 2A). Instead, the sequences representing the lowest quartile show a pronouncedly higher AA/TT/AT/TT content in the middle, which, we found was attributable to the higher poly (dA:dT) frequency (defined as a stretch of A or T of length 4 or above, Fig. 6C, bottom-left panel). This result is interesting as it is well known that poly (dA:dT) motifs cause stiffness of DNA sequences [6, 25, 29, 35]. We further analyzed the top/bottom quartiles of sequences from the Tiling library based on the $\hat{C}_0^s$ score, and again very similar results are observed (Fig. 6C, right panel). Lastly, we plotted the $\hat{C}_0^s$ score over all unique nucleosomes from the chemical map from [29], and the biotin-dependent oscillating pattern observed in ${{\hat{C}}_0}$ in Fig. 2C now disappears (Fig. 6D).

In summary, we conclude the newly defined smoothed intrinsic cyclizability score $\hat{C}_0^s$ can not only robustly remove the biotin tether bias but also more effectively reveal genuine DNA sequence features that affect DNA bendability.

### DNAcycP2

**Figure 7.**
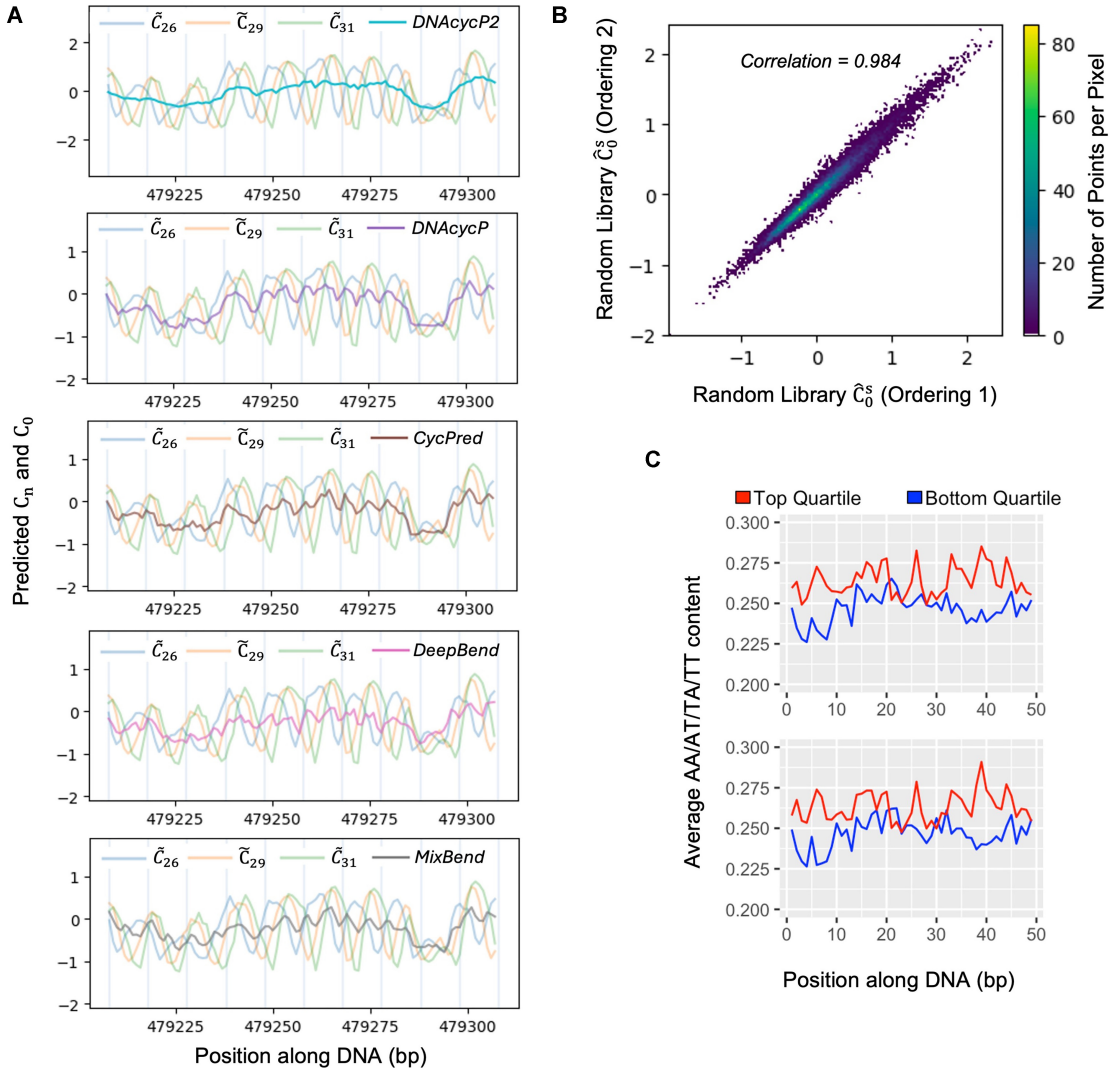
DNAcycP2 results and new estimate of intrinsic cyclizability ($\hat{C}_0^s$) calculated for the Random library. (**A**) Predicted intrinsic cyclizability using DNAcycP2, DNAcycP [25], CycPred [27], DeepBend [26], and MIXBend [28] (top to bottom) compared with predicted ${{C}_n}$ values (${{\tilde{C}}_n}$) on the example region of *S. cerevisiae* chromosome V in Fig. 1B. DNAcycP2 predicted values are displayed on the scale of normalized ${{\tilde{C}}_n}$ values, while the other models are on the scale of the unnormalized ${{\tilde{C}}_n}$ values for fair comparison. (**B**) Scatterplot of $\hat{C}_0^s$ for the sequences from the Random library by applying the local moving average method to concatenated sequences in two independent random orderings. All 50-bp sequences were connected into one artificial chromosome for each ordering. The ${{C}_n}$ values were predicted for every 50-bp sequence in the artificial chromosomes and then the moving average method was applied to generate the intrinsic cyclizablity estimate $\hat{C}_0^s$. Only the $\hat{C}_0^s$ values for the sequences which were part of the original Random library are plotted. (**C**) Average AA/AT/TA/TT content in the sequences from the Random library corresponding to the top and bottom quartiles of $\hat{C}_0^s$ for the first random ordering (top panel) and second random ordering (bottom panel). Only the $\hat{C}_0^s$ values for sequences which were part of the original Random library were considered.

DNAcycP2 follows the identical architecture of DNAcycP (Supplementary Fig. S1) but is trained using the smoothed intrinsic cyclizability score $\hat{C}_0^s$ for sequences in Tiling library. Notably, the correlation between the computed $\hat{C}_0^s$ values and the predicted intrinsic cyclizability score under DNAcycP2 on the yeast genome is 0.984 (Supplementary Table S3), much higher than the correlations between reported ${{\hat{C}}_0}$ and predicted intrinsic cyclizability from DNAcycP (Supplementary Table S4) and other tools, including CycPred [27], DeepBend [26], or MIXBend [28]. However, overall the predicted intrinsic cyclizability score from DNAcycP2 shows a good accordance with the originally reported ${{\hat{C}}_0}$ score, with correlation ranging from 0.75 to 0.90 for the four libraries (Supplementary Table S4). Compared with other existing models, DNAcycP2 better captures the underlying trend (Fig. 7A); while the predicted scores from the rest of all the tools exhibit more local oscillations that likely link to the residual biotin effect. Further, we extracted all 50-bp sequences from yeast chromosome V that correspond to the top and bottom quartiles of predicted intrinsic cyclizability score from DNAcycP2 and found no periodicity of AA/TT/TA/AT signal in either group (Supplementary Fig. S4A). Similar to what we observed using the computed $\hat{C}_0^s$, instead, we observe an association between elevated AA/AT/TA/TT content due to enriched poly (dA:dT) tracts in the middle of sequences for the bottom quartile (Supplementary Fig. S4A). Lastly, when aligned at the dyad of all unique nucleosomes, again, no oscillating pattern depending on DNA rotational angle was found in the predicted score (Supplementary Fig. S4B). These results suggest that DNAcycP2 was faithfully trained to yield the predictions that carry the properties of $\hat{C}_0^s$ as shown in Fig. 6.

To facilitate the use of DNAcycP2 by the research community, in addition to the Python package and web server, we developed an R package (to be available at Bioconductor). The new DNAcycP2 package also includes the prediction functions ported from DNAcycP for the convenience of comparison. We also improved the efficiency of these tools allowing for faster and less memory-intensive predictions. To compare the efficiency and runtime of the Python packages, we used a high-performance computer cluster. DeepBend does not allow for prediction from FASTA files, and MIXBend does not provide a prediction tool, so we will compare DNAcycP2 with the original DNAcycP and CycPred. Prediction times on the entire yeast genome (12.1 Mbp) are 64, 34, and 5 min for the original DNAcycP, CycPred, and DNAcycP2, respectively, each running with 36 GB RAM on 20 cores. Prediction times on chromosome I of the hg38 human genome (241 Mbp) are 22 and 1.5 h for the original DNAcycP and DNAcycP2, respectively, each running with 128 GB RAM on 20 cores. CycPred is omitted here as it is unable to predict on sequences with *N*s included (we attempted to predict on a modified version of the chromosome I reference genome with *N*s replaced by *A*s, but ran into memory issues even when we allocated 250 GB of RAM). For further reference, on a personal computer (16 GB RAM, M1 chip with 8-core CPU), prediction using the R package at full parallelization directly on the yeast genome FASTA file completes in 12 min, and on the hg38 human genome chromosome I FASTA file in just over 4 h. We hope that these developments allow for a broader, more flexible use of DNAcycP2 in intrinsic cyclizability prediction.

## Discussion

The high-throughput loop-seq assay opens a gate to quantify DNA cyclizability directly on thousands of sequences simultaneously and thus provides a unified way to study the association between DNA mechanics and various biological problems. There are a few technical issues in loop-seq assay that have not been fully addressed. First, the cyclizability quantified from loop-seq assay is a relative measure to other sequences in the given library, as between libraries, there is a library-specific constant difference. Thus, the quantified ${{C}_n}$ or ${{\hat{C}}_0}$ values in different libraries are not directly comparable in general. Furthermore, in practice, we are often interested in the DNA cyclizability or bendability of a single or a small amount of DNA sequences, performing the loop-seq experiment may become impractical. This makes it imperative to have a computational tool that can provide an accurate prediction of the DNA cyclizability under a unified measure (i.e. without library-specific difference). Following the loop-seq work, several tools, including our own work DNAcycP, have been developed. It was concluded that the DNA cyclizability is highly predictable, and the performance of these tools is very comparable. Second, the loop-seq assay requires attachment of biotin tether, the location of which dramatically biases the measured cyclizability score. Furthermore, this bias may also depend on the DNA sequence information, including AA/TT/TA/AT signals and other features through the amplitude term (i.e. ${{A}_n}( i )$ in Equation 1). However, the existing loop-seq data does not allow us to estimate ${{A}_n}( i )$ for each sequence without strong assumption. It becomes infeasible to quantify how biotin effect may depend on such sequence features. How to de-bias tether effect becomes a crucial step in yielding unbiased quantification of the intrinsic cyclizability. Basu *et al.* proposed the theoretical model in which the biotin effect depends on the phase angle of the DNA at the base pair where biotin is attached.

The first contribution of this paper was to train the prediction model on ${{C}_n}$ and verified the DNA helix phase angle-dependent biotin effect in the yeast genome by direct visualization. More importantly, we demonstrated that the residual biotin effect remains in the intrinsic cyclizability score ${{\hat{C}}_0}$ from two different perspectives. The first observation was that the most and least cyclizable sequences based on ${{\hat{C}}_0}$ (by quartiles) in the Tiling and ChrV libraries show strong dinucleotide periodic patterns, but antiphase. This similar pattern was also observed in the top/bottom quartiles of sequences in these two libraries based on ${{C}_{26}}$ value, likely suggesting the biotin effect in ${{C}_{26}}$ was partially carried over to ${{\hat{C}}_0}$. The periodic dinucleotide motif positioning has been shown in the literature for enhanced bendability, while phase change or shifting the sequence of a few base pairs with maintained periodicity should not dramatically undermine the sequence bendability. The oscillation pattern of the predicted intrinsic cyclizablilty due to DNA helix phase angle was also shown to have phase aligned with ${{\tilde{C}}_{26}}$ in the nucleosome sequences for all existing tools trained based on ${{\hat{C}}_0}$, again demonstrating the residual biotin effect.

The major contribution of our work is the proposed local moving average method to refine the estimates of intrinsic cyclizability, taking advantage of that the three libraries of loop-seq data came from the yeast genome. One particular possible caveat in the theoretical model (Equation 1) concerns the amplitude of the sine curve for the biotin effect. In practice, it is difficult to know what relationship ${{A}_n}$ should follow between $n$s. We showed in the real data that the ratio of ${{A}_n}^{\prime}s$ may vary dramatically between sequences, and in simulation, it was shown incorrect assumptions of the ratios may significantly undermine the estimation accuracy of the intrinsic cyclizability. The local smoothing method is relatively robust without making strong assumption of ${{A}_n}$. It is rooted in the biotin effect model in which the sine noise over a period (∼10.4 bp) can be averaged out to recover the intrinsic cyclizability. Indeed, through local smoothing, as we showed, the predicted/augmented ${{C}_n}$ values at different $n$s can lead to very consistent estimates of intrinsic cyclizability, and the average of the smoothed ${{C}_n}$ values results in further improvement of the estimates.

Lastly, the value of the proposed work is to provide an improved estimate of the intrinsic cyclizability score, the computed $\hat{C}_0^s$ shown to carry none or minimal biotin effect, that can be used to train the prediction models for DNA cyclizability.

However, it remains as an open question, for an arbitrary loop-seq library without a reference genome such as Random library, how can the local smoothing method be applied to calculate $\hat{C}_0^s$. To explore this, we generated two artificial chromosomes by concatenating all the 50-bp sequences from the Random library but in two random orderings. Using the deep-learning model for prediction of ${{C}_n}$, we obtained the augmented ${{\tilde{C}}_n}$ values at base-pair resolution in the entire artificial chromosomes. The intrinsic cyclizability estimates $\hat{C}_0^s$ of the original 50-bp sequences in the Random library from the local smoothing method show a high consistency (Pearson correlation = 0.984, Fig. 7B) between the two orderings. Plotting the AA/TT/AT/TA frequency in sequences corresponding to top and bottom quartiles of the $\hat{C}_0^s$ shows no periodic pattern in either case (Fig. 7C), suggesting the biotin bias was adequately removed. Lastly, the correlation between the computed $\hat{C}_0^s$ values from the two experiments and the predicted intrinsic cyclizability score under DNAcycP2 for the Random library is over 0.981. This suggests that although DNAcycP2 was trained using the $\ \hat{C}_0^s$ computed from the natural genome, it is effective to predict the intrinsic cyclizability of non-natural DNA sequences.

The proposed local smoothing method depends on the data augmentation of ${{C}_n}$ values and smoothing may reduce resolution. Although we showed ${{C}_n}$s are highly predictable using models trained on existing loop-seq data, better resolution loop-seq data with multiple replications is highly desirable for further investigation of the performance of the method proposed for estimation and prediction of intrinsic cyclizability.

## Supplementary Material

gkaf145_Supplemental_Files

## Data Availability

DNAcycP2 web server will be available at http://DNAcycP.stats.northwestern.edu for real-time prediction and visualization of C-score up to 20K bp, and a standalone Python package will be available for free download from https://github.com/jipingw. R/Python codes are also available at DNAcycP2-Python: https://doi.org10.5281/zenodo.14849705, DNAcycP2 R package: https://doi.org10.5281/zenodo.14849670.
