# Supplementary material for: DNAcycP2: improved estimation of intrinsic DNA cyclizability through data augmentation
Source: Nucleic Acids Res. 2025 Mar 12;53(5):gkaf145. doi: 10.1093/nar/gkaf145 (PMC11897897; doi:10.1093/nar/gkaf145)
Supplement: gkaf145_Supplemental_Files [file gkaf145_supplemental_files.zip › SupplementaryData.pdf]

SUPPLEMENTARY TABLES

		Test Library											
		Nucleosome			Random			Tiling			ChrV		
		$\tilde{C}_{26}$	$\tilde{C}_{29}$	$\tilde{C}_{31}$	$\tilde{C}_{26}$	$\tilde{C}_{29}$	$\tilde{C}_{31}$	$\tilde{C}_{26}$	$\tilde{C}_{29}$	$\tilde{C}_{31}$	$\tilde{C}_{26}$	$\tilde{C}_{29}$	$\tilde{C}_{31}$
Train Library	**Nucleosome**	0.893*	0.813*	0.808*	0.908	0.874	0.925	0.88	0.899	0.912	0.727	0.765	0.786
	**Random**	0.892	0.82	0.823	0.896*	0.869*	0.927*	0.871	0.894	0.91	0.721	0.762	0.787
	**Tiling**	**0.925**	**0.856**	**0.849**	**0.94**	**0.914**	**0.956**	**0.909***	**0.927***	**0.935***	**0.753**	**0.794**	**0.811**
	**ChrV**	0.914	0.84	0.843	0.92	0.895	0.947	0.895	0.917	0.93	0.737*	0.776*	0.798*

Supplementary Table 1. Correlations of predicted/augmented C_n values ($\tilde{C}_n$) with measured C_n values for each library. "*" indicates that column library was used for model training and the correlation was the average correlation from the test fold.

Random walk variance			Variance	Simulation Setting (i)		Simulation Setting (ii)		Simulation Setting (iii)	
C_0	A_{26}	log(a), log(b)	ε	$\bar{C}_{.,10.4}$	$\hat{C}_0$	$\bar{C}_{.,10.4}$	$\hat{C}_0$	$\bar{C}_{.,10.4}$	$\hat{C}_0$
1/144	1/25	1/100	1/16	0.995719	0.985087	0.996434	0.9294	0.996175	0.910147
1/144	1/25	1/100	1/4*	0.993296	0.944002	0.994106	0.894579	0.993828	0.877979
1/144	1/25	1/100	1/9*	0.995089	0.973929	0.995829	0.919961	0.995566	0.901485
1/144	1/25	1/100	1/25*	0.996011	0.99038	0.996715	0.933884	0.996457	0.91423
1/144	1/25	1/100	1/36*	0.99617	0.993291	0.996867	0.936357	0.996609	0.916464
1/144	1/25	1/36*	1/16	**	**	**	**	0.995762	0.909297
1/144	1/25	1/64*	1/16	**	**	**	**	0.996015	0.910108
1/144	1/25	1/144*	1/16	**	**	**	**	0.996241	0.910402
1/144	1/25	1/225*	1/16	**	**	**	**	0.996308	0.91089
1/144	1/9*	1/100	1/16	0.994265	0.985087	0.995952	0.930059	0.99576	0.912076
1/144	1/16*	1/100	1/16	0.995224	0.985087	0.996275	0.929526	0.996026	0.910818
1/144	1/36*	1/100	1/16	0.995997	0.985087	0.996529	0.929541	0.996255	0.909376
1/144	1/64*	1/100	1/16	0.996311	0.985087	0.996628	0.929789	0.996332	0.907933
1/64*	1/25	1/100	1/16	0.992387	0.984558	0.993121	0.927123	0.992856	0.907333
1/100*	1/25	1/100	1/16	0.994455	0.984603	0.995195	0.927318	0.994932	0.907561
1/225*	1/25	1/100	1/16	0.996692	0.985469	0.99739	0.931063	0.997136	0.912211
1/400*	1/25	1/100	1/16	0.997487	0.986168	0.998153	0.934106	0.997909	0.916061

Supplementary Table 2. Sensitivity analysis results. Correlations between the simulated ground truth C_0 values and the average of the smoothed C_n values ($\bar{C}_{.,10.4}$) and $\hat{C}_0$, the estimated C_0 values using Eq. 1, for various parameter choices across each simulation setting. "*" indicates a parameter value that is different from the original. "**" indicates that the change in parameter has no effect on the original simulation.

Test Library	Correlation
ChrI	0.984537218
ChrII	0.984129502
ChrIII	0.983683458
ChrIV	0.983780409
ChrV	0.983871867
ChrVI	0.983953062
ChrVII	0.984232966
ChrVIII	0.984085127
ChrIX	0.984368239
ChrX	0.984120696
ChrXI	0.984351565
ChrXII	0.983938966
ChrXIII	0.984104907
ChrXIV	0.984104412
ChrXV	0.983865345
ChrXVI	0.983982353

Supplementary Table 3. Correlations between DNAcycP2 predicted intrinsic cyclizability and new estimated intrinsic cyclizability score ($\hat{C}_0^s$) for each chromosome of S. cerevisiae.

		Model	
		DNAcycP2	DNAcycP
Test Library	Nucleosome	0.870295447	0.893
	Random	0.901267114	0.93
	Tiling	0.889888914	0.916*
	ChrV	0.746241685	0.773

Supplementary Table 4. Correlations of predicted intrinsic cyclizablity score using DNAcycP2 and DNAcycP with reported intrinsic cyclizability from Eq. 1 ($\hat{C}_0$) for each library.

SUPPLEMENTARY FIGURES

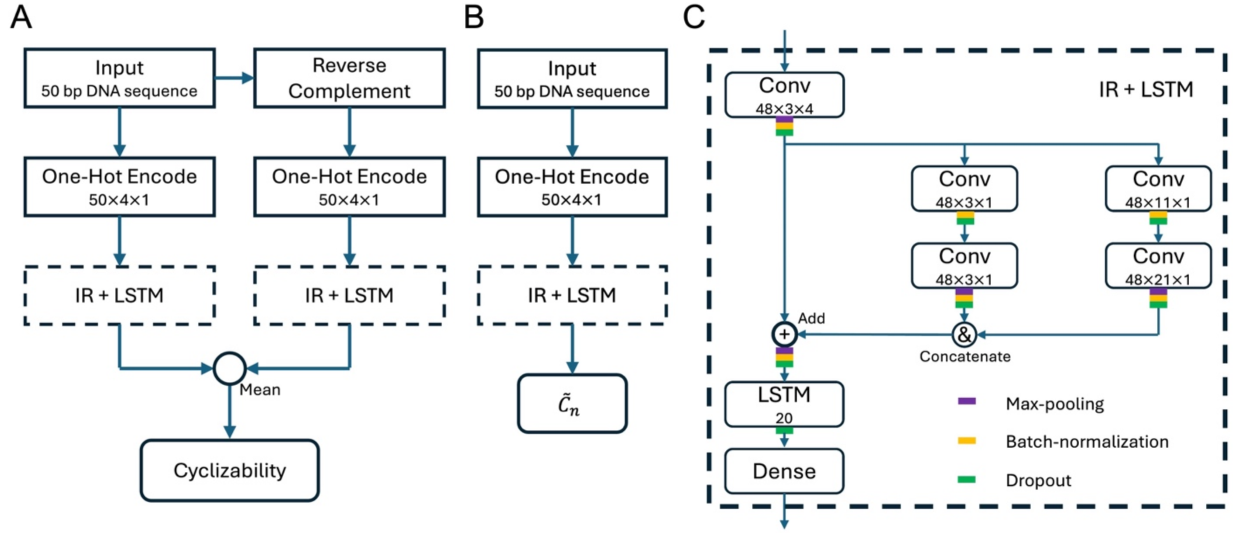

Supplementary Figure 1. Overview of model architecture. **(A)** Model architecture of DNAcycP2. **(B)** Model architecture of C_n predictors. **(C)** IR + LSTM block.

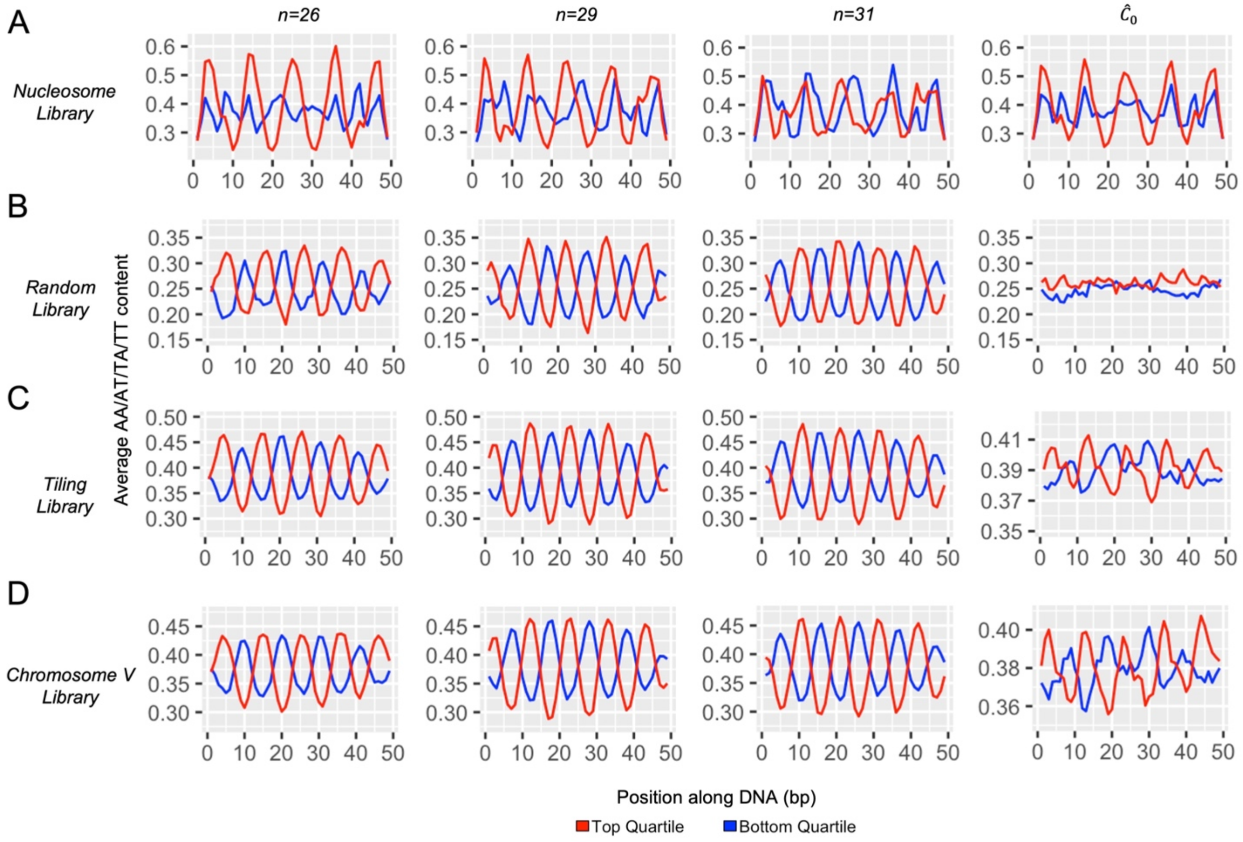

Supplementary Figure 2. The measured cyclizability score (C_n) from loop-seq and estimated intrinsic cyclizability score ($\hat{C}_0$) are biased by biotin locations. Average AA/AT/TA/TT content in the top and bottom quartiles of C_{26}, C_{29}, C_{31}, and $\hat{C}_0$ (left to right) in the four loop-seq libraries: **(A)** Nucleosome, **(B)** Random, **(C)** Tiling, and **(D)** ChrV. Note the change in scale on the y-axis for the furthest right plot in (C) and (D) to allow for easier comparison between plots.

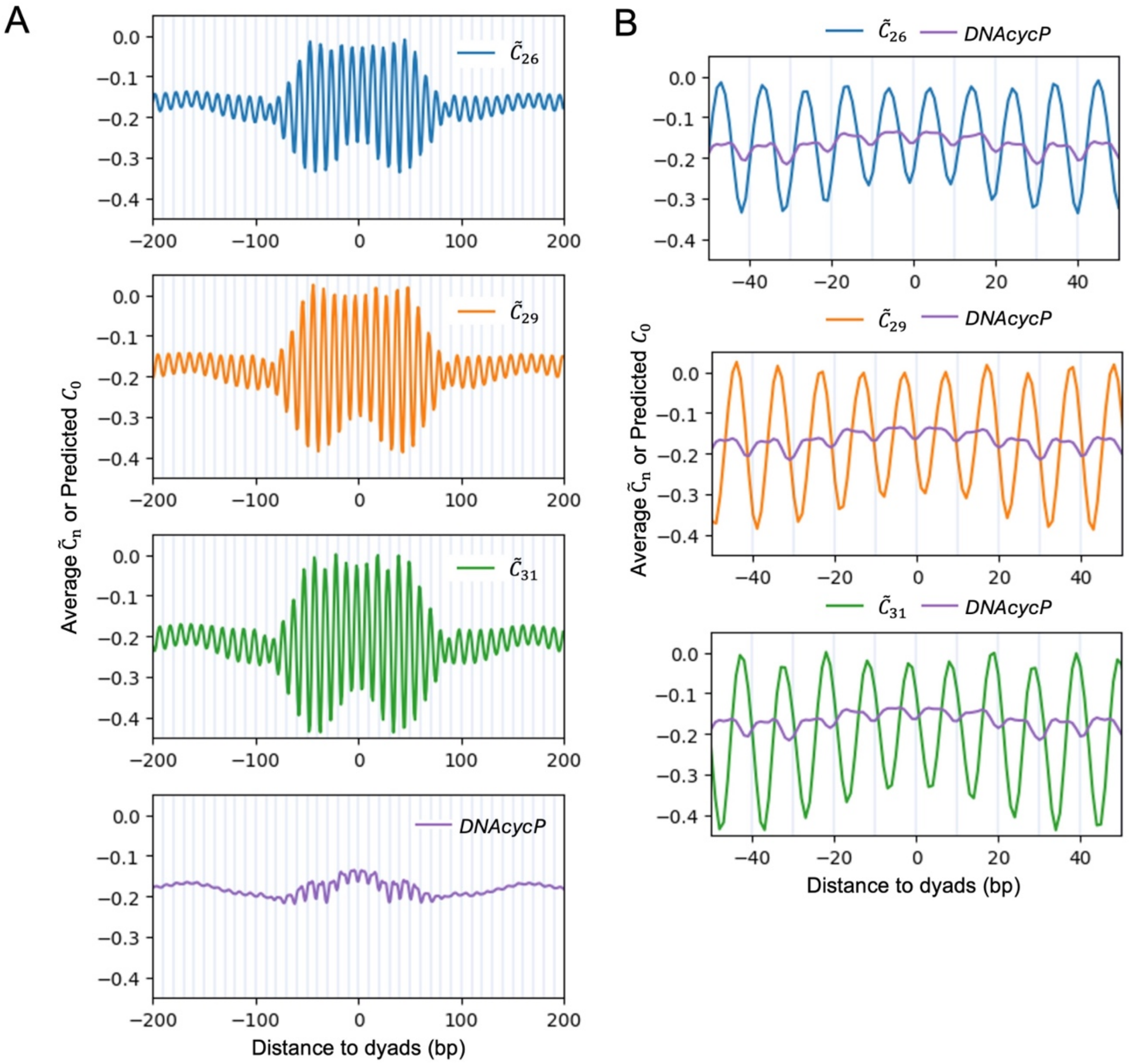

Supplementary Figure 3. Predicted intrinsic cyclizability score from DNAcycP shows a similar DNA phase angle dependent bias as $\tilde{C}_{26}$. **(A)** Mean unnormalized $\tilde{C}_{26}$, $\tilde{C}_{29}$, $\tilde{C}_{31}$, and DNAcycP predicted intrinsic cyclizability (top to bottom) aligned at all nucleosome dyads in the full genome of S. cerevisiae. **(B)** Zoomed-in version of (A) with DNAcycP predicted values overlayed on each panel.

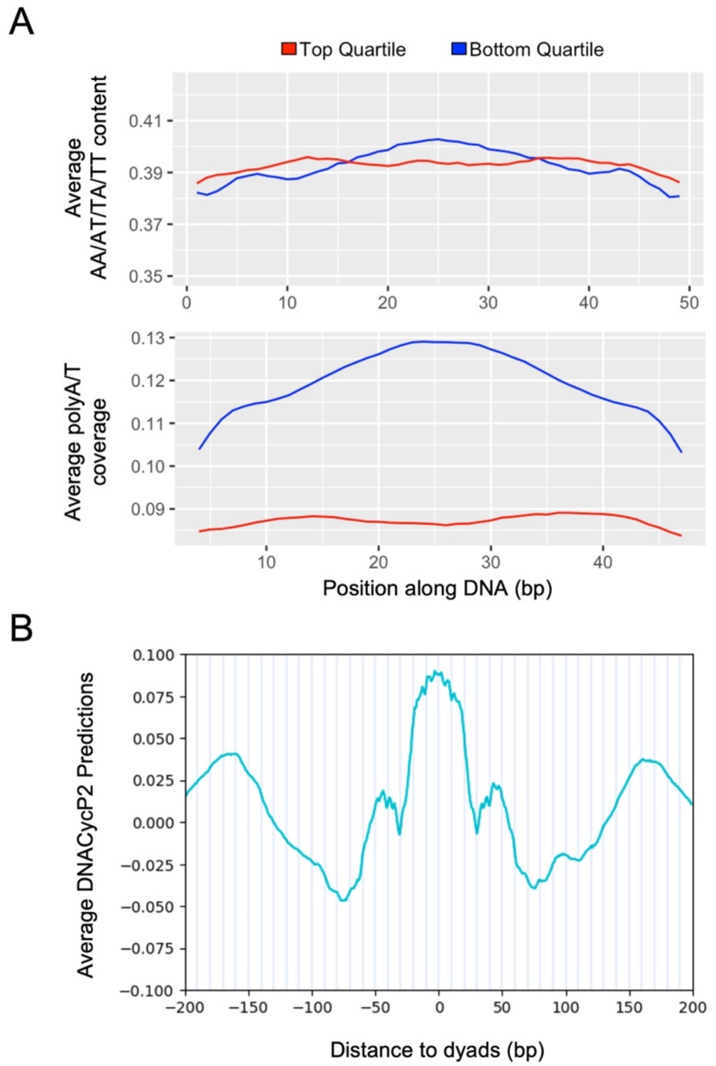

Supplementary Figure 4. (A) Average AA/AT/TA/TT content (top) and average poly(dA:dT) coverage (the proportion of sequences which have a stretch of A or T of length 4 or above at a given position) (bottom) in the top and bottom quartiles of DNAcycP2 predicted intrinsic cyclizability for every 50 bp sequence on S. cerevisiae Chromosome V. **(B)** Mean DNAcycP2 predicted intrinsic cyclizability values aligned at all nucleosome dyads in the full genome of S. cerevisiae.
